# Genome-Wide Characterization and Analysis of the *FH* Gene Family in *Medicago truncatula* Under Abiotic Stresses

**DOI:** 10.3390/genes16050555

**Published:** 2025-05-01

**Authors:** Jiatong Wang, Chunyang Zhou

**Affiliations:** College of Life Science and Technology, Changchun University of Science and Technology, Changchun 130012, China; 15731950256z@gmail.com

**Keywords:** formins, *Medicago truncatula*, abiotic stress

## Abstract

Background: The formin family proteins play an important role in guiding the assembly and nucleation of linear actin and can promote the formation of actin filaments independently of the Arp2/3 complex. As a key protein that regulates the cytoskeleton and cell morphological structure, the formin gene family has been widely studied in plants such as *Arabidopsis thaliana* and rice. Methods: In this study, we conducted comprehensive analyses, including phylogenetic tree construction, conserved motif identification, co-expression network analysis, and transcriptome data mining. Results: A total of 18 *MtFH* gene family members were identified, and the distribution of these genes on chromosomes was not uniform. The phylogenetic tree divided the FH proteins of the four species into two major subgroups (Clade I and Clade II). Notably, *Medicago truncatula* and soybean exhibited closer phylogenetic relationships. The analysis of cis-acting elements revealed the potential regulatory role of the *MtFH* gene in light response, hormone response, and stress response. GO enrichment analysis again demonstrated the importance of *FH* for reactions such as actin nucleation. Expression profiling revealed that MtFH genes displayed significant transcriptional responsiveness to cold, drought, and salt stress conditions. And there was a temporal complementary relationship between the expression of some genes under stress. The protein interaction network indicated an interaction relationship between MtFH protein and profilin, etc. In addition, 22 miRNAs were screened as potential regulators of the *MtFH* gene at the post-transcriptional level. Conclusions: In general, this study provides a basis for deepening the understanding of the physiological function of the *MtFH* gene and provides a reference gene for stress resistance breeding in agricultural production.

## 1. Introduction

In eukaryotic cells, the actin cytoskeleton is involved in many physiological processes, including cell polarization, cytokinesis, morphogenesis, cell motility, etc. [1,2,3]. Actin filaments, as one of the cytoskeleton components, can form a variety of array structures, such as thread-like bundle structures and branching actin filament network structures [4,5]. The elongation of actin filaments is achieved by adding actin monomers to the barbed end of actin filaments in a polarized manner. During the formation process, the nucleation process of actin monomer polymerization into a stable trimer core is the rate-limiting step [6]. At present, three nucleating agents—actin-related protein 2/3 (Arp2/3) complex, Spire, and formin—have been identified.

The Arp2/3 complex is a more fully characterized actin nucleation and recombination medium [7]. The Arp2/3 seven-subunit complex can be activated by binding to the nuclear factor Wiskott–Aldrich syndrome protein (WASP) to promote the synthesis of branched actin filaments and form a dendritic actin network [1,8]. Spire is a nuclear factor found in Drosophila and contains a WASP Homology 2 (WH2) domain. It contains four WASP homologous WH2 domains that promote the formation of actin filaments without Arp2/3 complexes [6,9]. Formin family proteins can also promote the formation of linear actin filaments independently of the Arp2/3 complex in some cases [7,10]. For example, in budding yeast, the formin protein Bni1 can stimulate the formation of actin filaments in vitro without the involvement of the Arp2/3 complex [7,11]. In the process of cell polarity formation, the actin cable formed by formin can act as a material transport track to ensure the supply of the required material for the establishment of polar parts [12]. During cell division, the actin cable formed by formin plays an important role in the correct orientation of mitotic spindles and the formation of contraction rings during cytokinesis [12,13,14].

Formin protein was first discovered in mice [15], and it is a group of important cytoskeletal regulatory proteins [16,17]. They play a key role in the dynamic assembly of actin filaments. Formin is not only involved in the polymerization of actin, especially at the exposed end of rapid growth, but also plays a direct regulatory role in the nucleation and polarization of non-branched filamentous actin structures [13,18,19]. Most formin proteins are characterized by the presence of a Formin Homology 2 (FH2) domain and a Formin Homology 1 (FH1) domain [20,21]. The FH1 domain contains continuous proline residues that can act as binding sites for profilin, which can bind to profilin and deliver G-actin subunits from the profilin–actin complex to the barbed end of filament growth, thereby increasing the elongation of the barbed end [7,22,23,24]. The FH2 domain is the most conserved part of the formin protein, with a length of about 400–500 amino acids, which is essential for formin to induce actin assembly in cells. According to previous studies, the FH2 domain is a dimer and continues to bind to the barbed ends of actin filaments while preventing cap proteins from binding to the barbed ends, which allows profilin–actin to be rapidly assembled into actin filaments [25,26]. In animals and fungi, the formin protein has a Formin Homology 3 (FH3) domain in addition to the FH1 and FH2 domains [27,28]. The FH3 domain is located at the amino terminus of the formin protein and consists of three blocks, and the FH3 domain is the most variable in the homologous region. Its main function is the localization of formin protein in cells [27].

In angiosperms, formin can be divided into two categories: class I and class II. The main features of class I formin proteins are a transmembrane domain in their N-terminal region and an extracellular domain rich in proline [29]. Class II formin protein contains a phosphatase and tensin homolog (PTEN)-like domain at the N-terminus [30]. In addition, class III formin proteins are currently only detected in plants containing flagellum sperm, which is characterized by the presence of a RhoGTP enzyme-activating protein (GAP) domain at the N-terminus [31].

At present, the main research on the formin family is concentrated in *Arabidopsis* and *Oryza sativa*. In *O. sativa,* studies of the *FH* gene family have revealed its importance in plant morphogenesis. Specifically, class I α protein OsFH1 can regulate root hair elongation [32]. Class II *FH* gene *OsFH5* regulates the overall morphology of rice at the cytoskeleton level by promoting actin polymerization, regulating actin filament dynamics, binding microtubules, and coordinating the spatial organization of microtubules and microfilaments [33]. In *Arabidopsis thaliana*, AtFH3 and AtFH5, two class I formin proteins, jointly regulate actin polymerization originating from the inner membrane of pollen tubes and control the construction of apical actin structure and vesicle transport [34]. AtFH8 can regulate the nucleation, elongation, and cutting of actin filaments through its FH1 and FH2 domains, and its FH1 domain can directly bind to profilin, which will lead to the development of root hair cells when it is overexpressed [35].

The *FH* gene family and its members have also been identified in plants such as soybean [36], potato [37], and wheat [18]. For example, most of the *FH* genes in soybean showed low expression levels under salt and ethylene stress, while *GmFH5*, *GmFH12*, *GmFH15*, and other genes showed increased expression under drought conditions. The expression of *TaFH* genes, such as *TaFH2* and *TaFH4*, was lower under low-temperature stress than in non-stress environments in the early stages of stamen development in wheat [18]. In leaf tissues of potato, the expression of *StFH1*, *StFH18*, and *StFH19* indicated their importance in plant drought tolerance [37]. These may imply that *FH* genes play an important role in plant growth and development for stress tolerance.

Legumes, as one of the main food sources for humans and animals, account for about one-third of the world’s crop yields today [38]. Legumes contain a large amount of protein and lipids, which are essential for organisms to absorb nutrients from plants [39]. At present, abiotic stresses such as salinity, drought, and cold caused by environmental changes have seriously affected the yield of crops [40]. Therefore, it is very important to study the genes that respond to abiotic stresses in legumes.

Compared with current mature model plants such as *Marchantia*, which has rapid reproduction, and the whole genome has been sequenced [41], *Medicago truncatula* also has model plant characteristics such as a completed whole-genome sequencing, high fruiting rate, high efficiency of genetic transformation, and fast seed regeneration [42]. In addition, *M. truncatula* has a high degree of genetic similarity with most legumes, and it can be used as a model plant of legume for the study of environmental resistance in order to be used to explore the resistance mechanism of legumes [43].

In this study, the formin gene family (*MtFH*) of *M. truncatula* was genome-wide identified, and its physical and chemical properties, motif structure, phylogenetic relationship, chromosome distribution, cis-acting elements, GO enrichment, MicroRNA (miRNA) prediction, and expression profile were analyzed. The results of this study not only help to reveal the function of *MtFH* in legumes but also provide new ideas for improving the resistance of *M. truncatula* to abiotic stress in agricultural production.

## 2. Materials and Methods

### 2.1. Identification and Physicochemical Properties Analysis of MtFH Gene Family

*M. truncatula*’s genome files, protein-sequence files, and annotation data are all taken from this website (https://medicago.legumeinfo.org/, accessed on 21 September 2024). The FH conserved domain’s (PF02181) was retrieved from the Pfam database [44] (http://pfam-legacy.xfam.org/, accessed on 21 September 2024). To identify MtFH proteins, we employed the HMM model in TBtools, filtering protein sequences with e-values below 0.05. To confirm the *MtFH* candidate gene, the conserved domain database (CDD) received the MtFH protein sequence. Ultimately, 18 MtFH proteins were identified. The ExPasy database (https://web.expasy.org/compute_pi/, accessed on 28 September 2024) [45] was used to examine the MtFH protein’s hydrophilicity, aliphatic amino acid index, isoelectric point, instability index, and quantity of amino acids. The Cell-PLoc 2.0 online program (http://www.csbio.sjtu.edu.cn/bioinf/Cell-PLoc-2/, accessed on 28 September 2024) was used to estimate the subcellular localization of MtFH protein [46].

### 2.2. Phylogenetic Tree of MtFH Gene Family and Analysis of Gene Structure, Domain, and Conserved Motifs

The FH protein sequences of soybean, *Arabidopsis*, and rice were obtained from the EnsemblPlants database (http://plants.ensembl.org/index.html, accessed 22 September 2024); and the FH members of soybean, *Arabidopsis*, and rice were extracted using the same methodology as that used to screen the FH family of *M. truncatula*; and the NJ method and MEGA 7.0 (version 7.0.26) were used to build the phylogenetic tree. A bootstrap value of 1000 was chosen. Then, we utilized the Evolview website (https://evolgenius.info//evolview-v2/#login, accessed on 26 September 2024) to beautify the outcomes. We analyzed conservative motifs using the MEME (http://meme-suite.org/tools/meme, accessed on 27 September 2024) website [47], and we set the maximum number of motifs to 10. The *MtFH* gene structure was extracted from the gene annotation file using TBtools. The CD-search online analysis website (https://www.ncbi.nlm.nih.gov/Structure/bwrpsb/bwrpsb.cgi, accessed on 27 September 2024) was used to assess and set the domain of the MtFH protein as an automatic parameter [47]. Finally, the individual results were visualized using TBtools (version 2.154), and the results were composited using Adobe Illustrator 2021.

### 2.3. Chromosome Localization, Collinearity, and Ka/Ks Analysis of MtFH Gene Family

Screening for information on the specific location of the *MtFH* gene in the thistledown *M. truncatula*, the distribution of *MtFH* gene in chromosome was visualized by TBtools software. Colinearity genes in the *FH* gene family were identified using the TBtools’ MCScanX plugin. The results were presented with TBtool visualization tools.

### 2.4. Analysis of Cis-Acting Elements of MtFH Gene Family

Cis-acting elements of the *MtFH* gene were analyzed by PlantCARE (https://bioinformatics.psb.ugent.be/webtools/plantcare/html/, accessed on 9 November 2024) using a 2000 bp sequence upstream of the *MtFH* gene. TBtools was used to visualize where a specific number of cis-acting elements bind in each gene, and heat maps were produced using these data. Finally, each result was synthesized on a single graph using Adobe Illustrator 2021.

### 2.5. MtFH Gene miRNA Prediction and GO Enrichment Analysis

The CDS sequence of *MtFH* gene was used to predict miRNA, and the expectation value in psRNAtarget (https://www.zhaolab.org/psRNATarget/, accessed on 23 February 2025) was set to 3.5. miRNA interactions with target genes were later visualized using Cytoscape (v3.10.0). GO enrichment analysis of MtFH protein was performed using the GO enrichment function of TBtools, and the enrichment results were submitted to the visualization platform (https://www.bioinformatics.com.cn/, accessed on 23 February 2025) for visualization.

### 2.6. Tissue-Specific and Stress-Related Expression Profiles of MtFH Gene Family

*MtFH* gene expression profile data in tissues were obtained at the website (https://medicago.legumeinfo.org/, accessed on 7 October 2024). The RNA-seq data of *M. truncatula* at 0 h, 2 h, 6 h, and 12 h under different stresses were downloaded from NCBI (https://www.ncbi.nlm.nih.gov/, accessed on 6 November 2024) [48]. Transcript data of *MtFH* gene were obtained by screening (Accession No.: GSM4056957, GSM4056956, GSM4056955, GSM4056954, GSM4056953, GSM4056952, GSM4056951, GSM4056950, GSM4056949, GSM4056948, GSM4056947, GSM4056946). The data were expressed as fragments (FPKM) per million mapped reads per thousand bases of transcription. Finally, the result data were visualized using the Heatmap tool of TBtools.

### 2.7. PPI Interaction Network

Protein interaction networks were constructed using the STRING 11.5 (https://string-db.org/, accessed on 21 November 2024) [49] with parameters set to medium confidence. Eighteen MtFH proteins were used to construct relational networks with five other proteins.

## 3. Results

### 3.1. Identification and Information of FH Gene Family in M. truncatula

The identification of the whole genome of *M. truncatula* (Table 1) showed that there were 18 *MtFH* genes in *M. truncatula*, which were expressed as *MtFH01* to *MtFH18*. The table shows that the protein length was 689 to 1928 amino acids, the relative molecular weight was 77.1 to 206.92 kDa, and the isoelectric point was 5.84 to 9.21. The instability coefficient is concentrated between 45 and 69, and the instability coefficient of *MtFH05* reaches 80.43. The aliphatic amino acid coefficient is between 72 and 81, while the GRAVY (grand average of hydropathicity) is negative. The subcellular localization prediction results indicate that the majority of MtFH proteins are found in the nucleus, whereas *MtFH03* and *MtFH04* are expected to be found in the chloroplast, and only *MtFH18* may be found in the vacuole.

### 3.2. Chromosomal Distribution of FH Gene Family in M. truncatula

**Figure 1:**
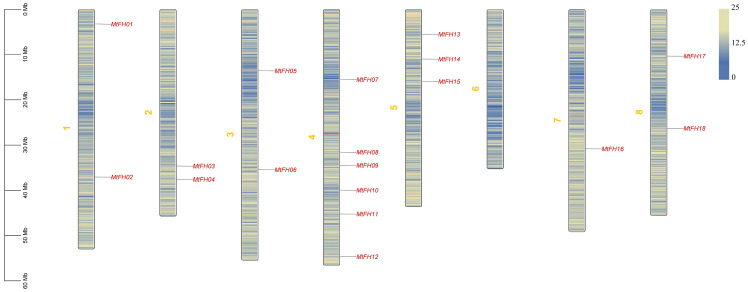
The position distribution of *MtFH* gene family in chromosomes. The scale on the left is used to measure the length of chromosomes, and The density of genes in each chromosome is shown in blue and yellow colors.The yellow numbers to the left of each chromosome represent each chromosome.

According to the statistics for *MtFH* gene distribution on chromosomes, 18 *MtFH* genes have been marked on eight chromosomes of *M. truncatula* (Figure 1). Among them, there are two *MtFH* genes on chromosomes 1, 2, 3, and 8; three *MtFH* genes on chromosome 5; and only one *MtFH* gene on chromosome 7. It is worth noting that there are six genes—*MtFH07*, *MtFH08*, *MtFH09*, *MtFH10*, *MtFH11*, and *MtFH12*—on chromosome 4, while there is no *MtFH* gene on chromosome 6. This indicates that the *MtFH* gene is widely and unevenly distributed in the chromosomes of *M. truncatula*.

### 3.3. Phylogenetic Analysis of FH Family in M. truncatula

**Figure 2:**
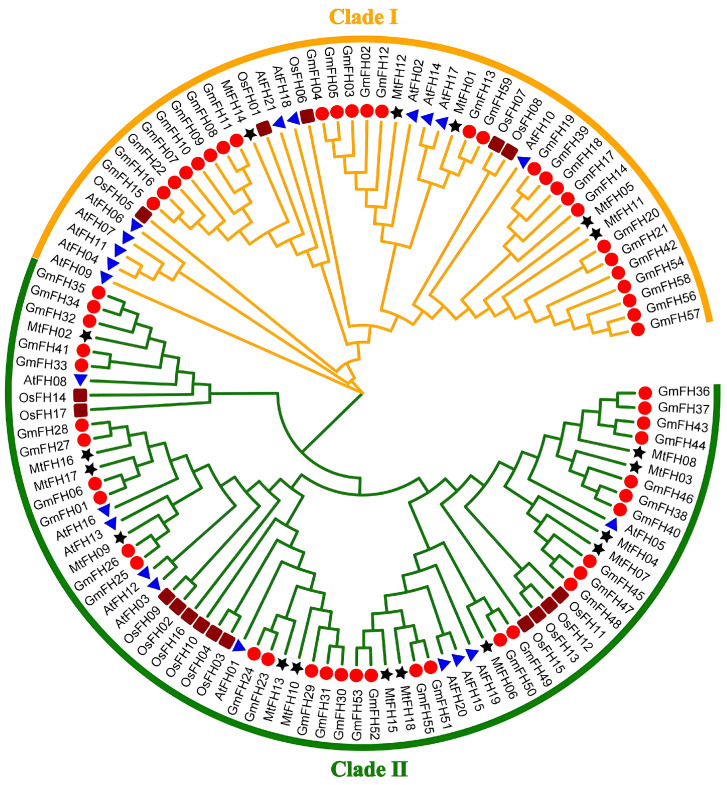
Phylogenetic tree of FH among different species. ★ represents FH members of *M. truncatula*, 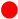 represents FH members of *Glycine max*, 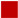 represents FH members of *Oryza sativa*, 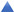 represents FH members of *Arabidopsis thaliana*.

We made a phylogenetic tree using the FH proteins of four species: *O. sativa* and *A. thaliana* are two model plants, and *Glycine max* and *M. truncatula* are two legume plants (Figure 2). The phylogenetic tree was split into two groups, Clade I and Clade II. The distribution of MtFH members in the two groups was uneven, with five MtFH members in Clade II and 13 MtFH members in Clade I. Also, the fact that most of the MtFH members and GmFH members were in one branch showed that the *FH* gene family of *M. truncatula* was very similar to the soybean *FH* gene family.

### 3.4. Evolutionary Relationship, Motif, Conserved Domain, and Gene Structure Analysis of FH Gene Family in M. truncatula

**Figure 3:**
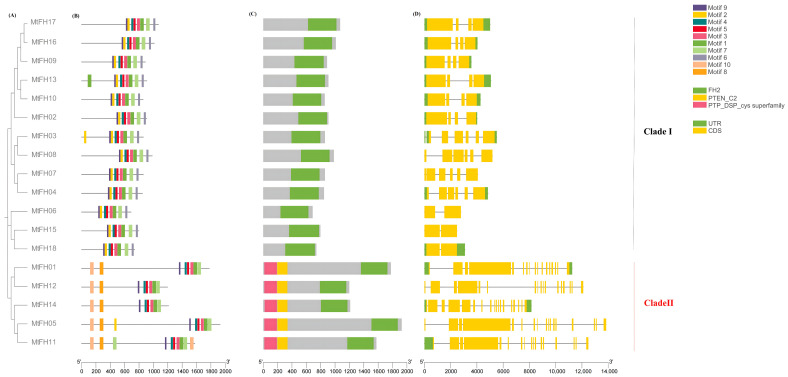
The evolutionary classification, conserved motifs, conserved domains, and gene structure of *MtFH*. (**A**) The phylogenetic tree of MtFH. (**B**) The conserved motif of MtFH. Each different colored rectangular box represents a conserved motif. (**C**) The conserved domain of MtFH protein. (**D**) The gene structure of *MtFH*. The yellow rectangles are exons, the green parts are the untranslated regions of the two segments, and the remaining black lines indicate introns.

Analysis of the motifs of 18 *MtFH* gene family members (Figure 3) showed that the family members can be divided into two main subgroups, Clade I and Clade II (A), each member containing eight to 10 motifs. The vast majority of *MtFH* members in Clade I contain eight motifs; only *MtFH13* and *MtFH03* contain nine motifs. In Clade II, *MtFH01, MtFH12*, and *MtFH14* contained eight motifs, *MtFH05* contained nine motifs, and *MtFH11* contained 10 motifs (B). In addition, through the analysis of conserved domains, it was found that the *FH* family has a typical domain, namely, FH2. All *FH* members in Clade I contain only the FH2 domain, while all *FH* members in Clade II contain the PTEN_C2 domain and the PTP_DSP_cys superfamily domain in addition to the FH2 domain (C). According to the gene structure study, the *MtFH* gene was unevenly lengthy, with *MtFH05* having the longest length and *MtFH15* having the shortest. The 5’ and 3’ UTR regions were discovered in most *FH* members, but there was no UTR region in *MtFH08*, *MtFH07*, *MtFH06*, *MtFH15*, *MtFH12*, or *MtFH05.* It is important to mention that *MtFH11* only includes 5’ UTR (D).

### 3.5. Intermediate Collinearity Analysis of M. truncatula

**Figure 4:**
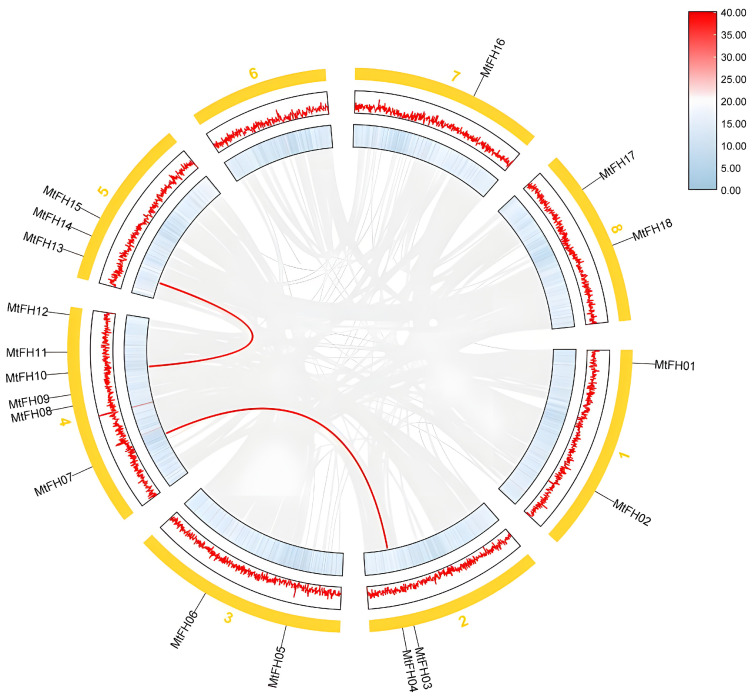
Intraspecific collinearity analysis of *M. truncatula*. The yellow rectangle represents the chromosome, and the gene pairs with segment repetition are shown by the red line. The gray lines represent collinearity genes across the genome. The middle and inner rectangles are two different representations of gene density. The yellow numbers represent the each chromosome.

In order to study the distribution of duplicated genes on each chromosome, we analyzed the replication of *MtFH* gene family members (Figure 4). The results showed that the *MtFH* gene contained two fragment repeat pairs, and no tandem repeat pairs were found. We also examined the evolutionary rate of gene sequences by computing the value of Ka/Ks (Table 2). The two gene pairs’ Ka/Ks values were all less than 1, which suggests that purification selection had occurred during evolution and that these genes were more likely to retain their original functions.

### 3.6. Collinearity Analysis Between M. truncatula and Other Plants

**Figure 5:**
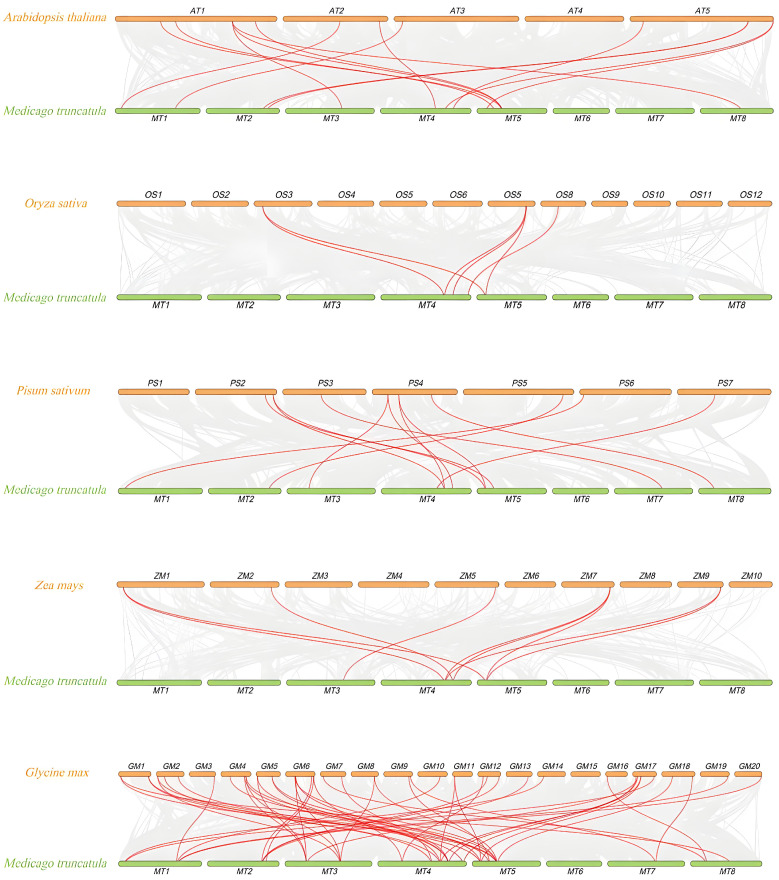
Collinearity analysis of *FH* genes between *A. thaliana*, *O. sativa*, *P. sativum*, *Z. mays*, *G. max*, and *M. truncatula*. The grey line is the collinearity between the genomes of the two species. The red line is the collinearity of *FH* gene.

We analyzed the collinearity between five different species and *M. truncatula* (Figure 5). The results showed that the 14,12,12,9, and 50 *MtFH* genes were collinear to genes in *Arabidopsis*, rice, pea, maize, and soybean genomes (Table S1). Among them, there were the most collinear pairs between *M. truncatula* and soybean. Furthermore, the *MtFH13*, *MtFH11*, and *MtFH10* genes have orthologous counterparts in five different species, which suggests that these three genes are evolutionarily conserved and relatively important.

### 3.7. Analysis of the MtFH Gene’s Cis-Acting Elements

**Figure 6:**
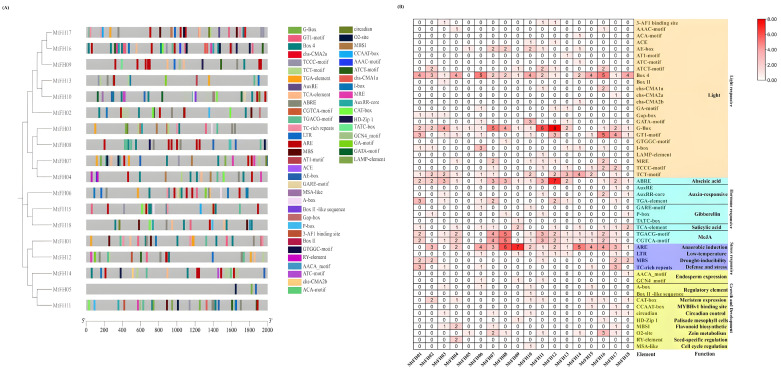
(**A**) Distribution of cis-acting elements in each *MtFH* gene. (**B**) Quantitative distribution of each cis-acting element in *MtFH* gene and their functional classification.

The cis-acting elements of the *MtFH* gene were characterized (Figure 6). Among them, *MtFH05* contains only three cis-acting elements, while *MtFH16* contains the largest number of elements, 38. Most *MtFH* genes contain 22–33 cis-acting elements(A). The 50 elements were divided into four categories: light-response elements, hormone-response elements, stress-response elements, and growth- and development-related elements. Among them, the light-response element contains 24 kinds. Box 4, G-Box, and TCT-motif elements are the representative elements in *MtFH* genes: 88% of *MtFH* genes contain Box 4 and G-Box elements, and 66% of *MtFH* genes contain TCT-motif elements. The hormone-response element contains 10 elements, such as the auxin-response element, salicylic acid-response element, gibberellin-response element, abscisic acid-response element, and methyl jasmonate-response element. It is worth noting that 83% of *MtFH* genes contain abscisic acid-response elements, while 72% of *MtFH* genes contain methyl jasmonate-response elements. There are four stress-response elements, including anaerobic induction element (ARE), low-temperature stress element (LTR), drought stress element (MBS), and defense and stress element (TC-rich repeat). It is worth mentioning that 83% of *MtFH* genes contain anaerobic-inducible elements, especially *MtFH09* and *MtFH08*. The growth- and development-related element contains 12 elements, of which the seed-specific regulatory element (RY-element) is only present in MtFH04, and the cell cycle regulatory element (MSA-like) only exists in *MtFH10* (B). According to the findings, the *MtFH* gene family is essential for hormone and stress responses, as well as for plant growth and development.

### 3.8. Prediction of miRNAs for MtFH Gene

**Figure 7:**
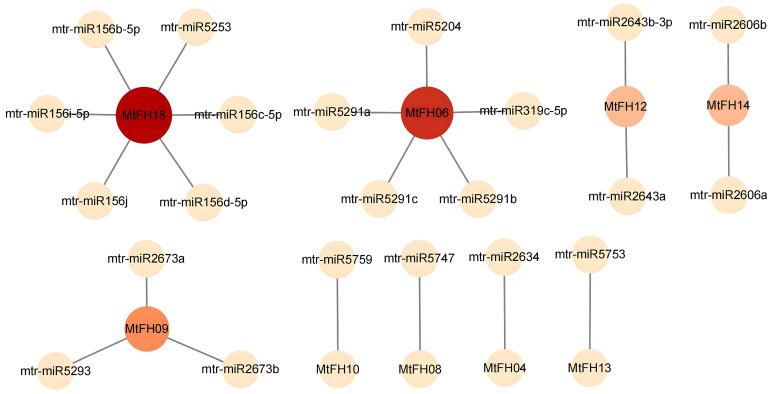
Diagram of the predicted miRNA interaction network with *MtFH* gene. Both the color and size of the nodes are proportional to the Degree Centrality value; the larger the degree value, the darker the node color and the larger the node size.

We performed miRNA prediction for the *MtFH* gene using the psRNATarget website and identified 22 miRNAs (Figure 7), which targeted *MtFH18*, *MtFH06*, *MtFH09*, *MtFH12*, *MtFH14*, *MtFH10*, *MtFH08*, *MtFH04*, and *MtFH13*. Among them, the number of miRNAs targeting *MtFH18* was the highest, totaling six. *MtFH10*, *MtFH08*, *MtFH04*, and *MtFH13* were all targeted by one miRNA. Each miRNA targeted only one gene, and all of these miRNAs acted as shear genes.

### 3.9. GO Enrichment Analysis

**Figure 8:**
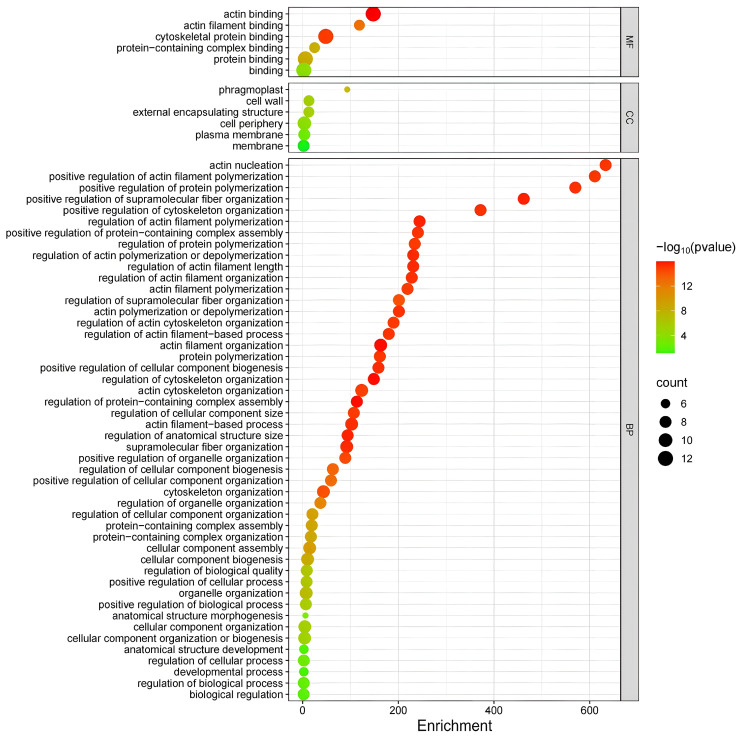
GO enrichment plot of *MtFH*. The vertical axis is the GO annotation, the horizontal axis is the fold enrichment, the color of the dots symbolizes the significant degree of gene enrichment, and the size of the dots indicates the number of genes.

The *MtFH* genes were annotated using GO ontology annotation (Figure 8), and the number of annotations belonging to Biological Process was the largest, totaling 48. Molecular Function and Cellular Component included the same number of GO annotations, both of which were six. Specifically, the annotations in Biological Process mainly covered the reactions of “actin nucleation”, “cytoskeleton regulation”, and “actin filament elongation”, while the annotations in Cellular Component mainly included the location information of the *MtFH* gene product such as the cell wall, plasma membrane, membrane-forming body, and external encapsulation structure. In Molecular Function, the number of *MtFH* genes in the “actin-binding” and “cytoskeletal protein-binding” functional annotations was high and significantly enriched. These results indicate that *FH* has an important role in binding to actin and the cytoskeleton.

### 3.10. Analysis of Expression Patterns of MtFH Gene in Different Tissues of M. truncatula

**Figure 9:**
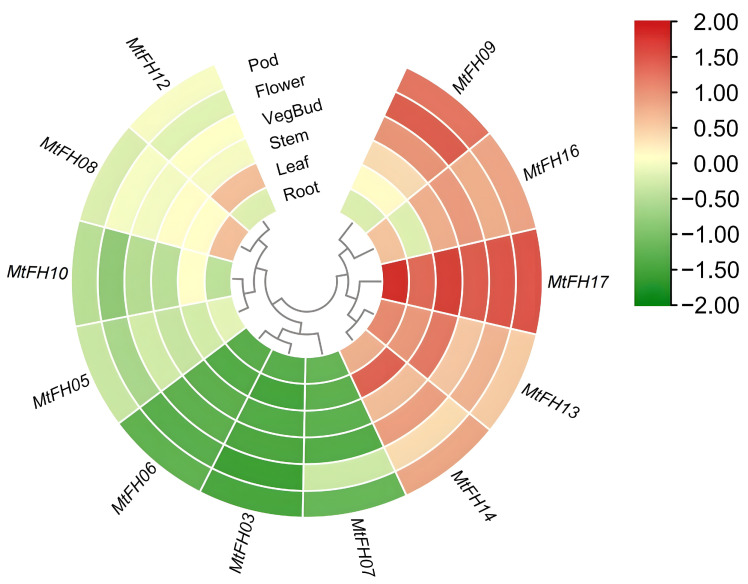
Heatmap of *MtFH* gene expression between six different tissues, the colors in the graph from green to red indicate small to large expression, and the genes were clustered according to their expression.

In order to further study the function of the *FH* gene in the growth and development of *M. truncatula*, we analyzed the expression level of the *MtFH* gene family in different tissues (Figure 9). The expression levels of 12 *MtFH* genes (six *MtFH* genes were not found in the expression profile) in six tissues of root, leaf, stem, vegbud, flower, and pod were measured. Among them, *MtFH17* has the highest expression level in all six tissues, and *MtFH13* and *MtFH14* were expressed in almost all tissues. *MtFH09* was expressed at a high level in vegbud, flower, and pod, and *MtFH16* was expressed a at high level in root, stem, vegbud, flower, and pod tissues. In addition, the expression of *MtFH10*, *MtFH05*, *MtFH06*, *MtFH03*, and *MtFH07* was at a low level. Notably, the expression of *MtFH12* was up-regulated only in leaf, while *MtFH08* was up-regulated only in root.

### 3.11. Expression Pattern of MtFH Gene Under Abiotic Stress

**Figure 10:**
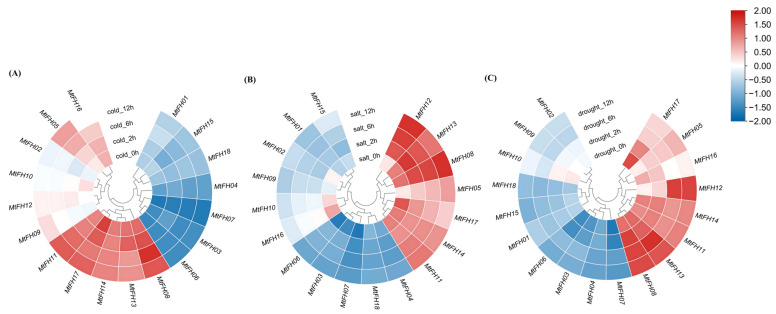
Heatmap of *MtFH* gene expression at 0 h, 2 h, 6 h, and 12 h under different stress conditions. (**A**) The expression profile of *MtFH* gene at four time points under cold stress. (**B**) The expression profile of *MtFH* at four time points under salt stress. (**C**) The expression profile of *MtFH* at four time points under drought stress.

We evaluated the expression level of the *MtFH* gene under different stress conditions and different action times (Figure 10). In cold stress, *MtFH08*, *MtFH13*, *MtFH14*, *MtFH17*, and *MtFH11* had high expression levels, and *MtFH06*, *MtFH03*, *MtFH07*, *MtFH04*, *MtFH18*, *MtFH15*, and *MtFH01* had lower expression. Notably, *MtFH16* was expressed at 0 h, 2 h, and 6 h and down-regulated at 12 h. The expression of *MtFH05* and *MtFH09* showed a sequential up-regulation from 0 h to 12 h, whereas the expression of *MtFH10* showed a decreasing trend from 0 h to 6 h (A). In salt stress, *MtFH12* and *MtFH08* showed a significant increase in expression from 0 h to 2 h and were at a high level of expression from 2 h to 12 h. *MtFH13* showed an increase in expression from 0 h to 2 h but a decreasing tendency from 2 h to 12 h. *MtFH05* showed an increasing tendency in expression, while *MtFH17* showed a decreasing tendency. *MtFH14* and *MtFH11* expression was stable and at a high level. *MtFH06*, *MtFH03*, *MtFH07*, *MtFH18*, and *MtFH04* had lower expression. In addition, *MtFH02*, *MtFH10*, and *MtFH16* had some expression at 0 h, and all showed down-regulation of expression at subsequent times (B). Under drought stress, *MtFH14*, *MtFH11*, *MtFH13*, and *MtFH08* had higher expression, while *MtFH06*, *MtFH03*, *MtFH04*, *MtFH07*, *MtFH01*, *MtFH15*, and *MtFH18* had lower expression. In addition, the expression of *MtFH17*, *MtFH16*, *MtFH09*, and *MtFH02* showed a down-regulation trend from 0 h to 12 h, while the expression of *MtFH05* continued to increase from 0 h to 12 h. Notably, *MtFH10* had a certain expression at 0 h and 2 h and a significant decrease in expression at 6 h. *MtFH12* had a relatively low expression at 0 h and 2 h and a significant increase in expression at 6 h (C).

### 3.12. Analysis of Protein–Protein Interaction Network

**Figure 11:**
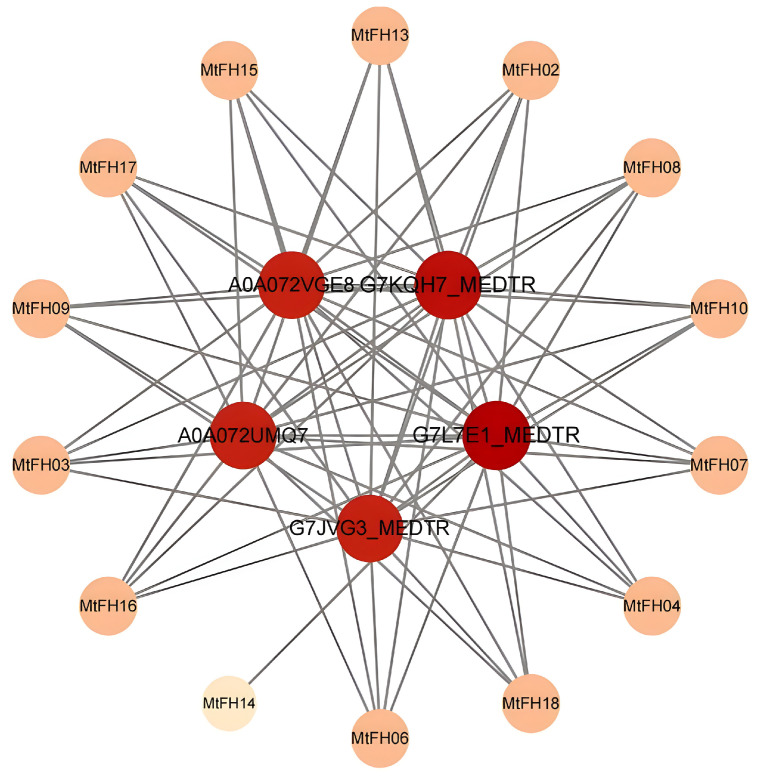
The interaction network between MtFH protein and other proteins.

The interaction network between the MtFH protein and other proteins was constructed based on the STRING database (Figure 11). There are 14 MtFH proteins associated with the other five proteins; only MtFH05, MtFH11, MtFH01, and MtFH12 have no interaction relationship. It is worth noting that five other proteins belong to the profilin, transcription factor Znf-LSD family, Muniscin carboxy-terminal mu-like domain protein, and Transmembrane protein, respectively. These proteins are mainly involved in biological processes: actin cytoskeleton organization (GO:0030036), actin filament organization (GO:0007015), actin nucleation (GO:0045010), etc. This matches the findings of earlier studies; that is, the interaction between formin protein and profilin promotes the nucleation and elongation of actin filaments, which, in turn, plays a regulatory role in the actin cytoskeleton [50]. These contents are helpful for studying the function and regulation mechanism of the FH protein.

## 4. Discussion

Formin proteins are widely present in plants. By participating in the process of actin assembly and nucleation, they lead to the formation of different cytoskeleton structures, which indirectly affect cell shape and function [51,52]. At present, the formin protein family has been identified in a variety of plants. In *Arabidopsis* and rice, 21 and 16 *FH* genes have been identified, respectively. In the following investigation, 26, 34, and 25 *FH* genes were found in potato, soybean, and wheat, respectively. However, the formin gene family in *M. truncatula* has not yet been completely discovered. Therefore, this research fully studied and identified the formin gene in *M. truncatula*.

*MtFH* genes are unevenly distributed on seven chromosomes except chromosome 6, which may be related to the enrichment of fragment replication events on specific chromosomes. In the analysis of physical and chemical properties, the length and molecular weight of MtFH protein are highly variable, and the isoelectric point of most MtFH proteins is between 5 and 9. In addition, the aliphatic index of MtFH protein is between 62 and 86, indicating that the content of non-polar amino acids in the protein may be higher. The GRAVY values were negative, indicating that MtFH proteins were hydrophilic proteins [53].

Phylogenetic analysis of FH2 domain sequences from four diploid plants resolved the formin family into two clades (I and II). Notably, soybean and *M. truncatula* FH proteins clustered more closely than those of Arabidopsis or rice, reflecting their shared legume ancestry and high homology. Structural characterization further supported this division: all MtFH members harbor a conserved FH2 domain, with Clade II proteins exhibiting additional motifs (8–10 per protein). Similar domain architectures in Arabidopsis and rice formins underscore the evolutionary conservation of this gene family across angiosperms.

Gene duplication can lead to the generation of diverse functional genes, thereby promoting the evolution of organisms [54]. We found three fragment repeat pairs in the *MtFH* gene family, but no tandem repeats were found. The Ka/Ks values of these gene pairs were calculated, and the results were all less than 1. This is consistent with the results in the potato study, where all seven pairs of genes were subjected to purifying selection [37], indicating that the *MtFH* gene family may have undergone purification selection and that these genes may be more conservative within the species, ensuring the stability of organism evolution, while fragment duplication may also be one of the reasons for genome amplification. Collinearity analysis revealed stronger synteny between *M. truncatula* and *G. max* than with four other species, reflecting a conserved gene structure and function within legumes.

The cis-acting elements of the *MtFH* gene may be linked to light-response and growth- and development-, stress-, and hormone-related processes [55,56]. It is worth mentioning that ABRE-response elements and MeJA-response elements are more distributed in *MtFH* genes. The same is true for the cis-acting element study of the soybean FH gene, where MeJA-responsive elements and ABRE were distributed in high numbers [36]. ABRE-response elements regulate the expression of downstream genes by binding to ABRE-binding factors (AREB/ABF), thus playing a key role in promoting plant response to adversity in ABA signaling [57]. It has been confirmed that ABA plays an important role in response to high salt, drought, and other abiotic stress environments [58,59,60]. MeJA is crucial for the growth of plants, seed germination, and senescence and also has a strong response to salt stress. Previous studies have confirmed that MeJA can promote plant growth and development by reducing the inhibition of photosynthesis by salt stress [61,62]. This study shows that the *MtFH* gene is crucial for regulating salt stress. In addition, there are some other types of elements in the cis-acting elements of the *MtFH* gene, such as ARE (anaerobic-induction element), MBS (drought-response element), LTR (low-temperature-response element) and so on, which highlights the importance of the *MtFH* gene in plant response to abiotic stress.

miRNA is a kind of tiny RNA that can bind with the 3’UTR region of mRNA through base complementary pairing to inhibit the process of translating proteins or cutting mRNA, and it is mainly involved in the post-transcriptional expression regulation of genes [63]. An miRNA can regulate the expression of one or more genes, and, similarly, a gene can be regulated by one or more miRNAs [64]. According to previous reports, the miR156 family targets various *StFH* genes in potato studies, and miR156 has been shown to improve drought tolerance in alfalfa plants by repressing gene expression [65]. In that study, the miR156 family targeted the *MtFH18* gene, while *MtFH18* expression was low under drought stress, which was hypothesized to be a possible inhibitory effect of miR156 on the target gene; miR319 targets *MtFH06* and may act as a homeostatic factor after drought injury, and this role is conserved between plants such as *Medicago ruthenica* and *M. truncatula* [66]. In another study, the expression of *MtFH12* was higher in salt stress, and miR2643 targeted *MtFH12*; it was hypothesized that *MtFH12* might respond to salt stress through the regulatory effect of miR2643, which corresponded to the previous study that miR2643 responded to salt stress in *M. ruthenica* [67].

In the GO enrichment analysis, *MtFH* genes were significantly enriched in the processes and functions of actin nucleation, the regulation of cytoskeleton, and actin binding, which demonstrates that formin acts as an actin nucleating agent, promotes actin nucleation, extends actin filaments, and regulates the cytoskeleton.

The expression of *MtFH17*, *MtFH14*, and *MtFH13* in all six tissues was at a high level, which indicates that these genes might be key as core regulatory genes for cellular functions during plant growth and development. Some genes were only highly expressed in one tissue, such as *MtFH08* in roots compared with other tissues, which suggests that *MtFH08* is closely related to root growth, similar to *AtFH8* in *Arabidopsis* and *OsFH1* in rice, which can regulate the growth of root hair cells.

Abiotic stresses, which mainly include salt stress, drought stress, and low-temperature stress, are the main limiting factors affecting crop yield and planting area. It has been shown that the expression of the *AtFH5* gene increased in the root system under salt stress. In this study, we found that *MtFH12*, *MtFH08*, and *MtFH13* had a significant response under salt stress. *MtFH17*, *MtFH16*, *MtFH08*, *MtFH11*, *MtFH14*, and *MtFH13* had a higher expression in early salt stress, which is consistent with the conclusion that most of the *GmFH* genes in soybeans have higher expression levels in the early stage under salt stress [36], which might be implicated in the early response mechanism of *M. truncatula* in response to salt stress adversity. Previous studies have shown that the regulation of stomatal conductance is one of the main ways for *M. truncatula* to cope with drought stress [43], and the expression of *StFH18* and *StFH19* in leaf tissues was higher and enhanced drought tolerance in potato [37]. In the present study, *MtFH13* and *MtFH14* are highly expressed under drought stress and in leaf tissues, which implies that these genes may respond to drought stress by participating in the regulation of leaf stomata. Similar to that in salt stress, the expression of *MtFH17*, *MtFH16*, and *MtFH10* started to decrease significantly after 2 h of drought treatment, while that of *MtFH12* increased significantly after 2 h; this implies that some of the same mechanisms exist between drought resistance and salt resistance, which is the same conclusion as in previous studies [43]. Under cold stress, studies on cotton (*Gossypium Raimondii*) showed that the expression of *GrFH4*, *GrFH6*, *GrFH11*, *GrFH12*, *GrFH14*, and *GrF21* was up-regulated. In the present study, *MtFH17*, *MtFH13*, *MtFH11*, *MtFH14*, and *MtFH08* had a strong response under cold stress. *MtFH17*, *MtFH13*, and *MtFH11* all contained low-temperature-response elements (LTRs), which is consistent with the result that the response of *GrFH21* in *G. Raimondii* under cold stress mostly appeared in LTR motifs [68]. These genes, such as *MtFH05* and *MtFH17*, were expressed in the late and early stages of stress under salt and drought stress, respectively, which was similar to the expression of the two genes, *GmFH10* and *GmFH29*, that were specifically expressed at 24 h and 4 h under salt stress, respectively. This suggests that there is a dynamic spatiotemporal regulatory property in soybean, which may hypothetically facilitate the plant’s stage of regulation of stress resistance [36].

We reviewed examples of previously screened genes. For example, in terrestrial cotton, *GhFH20* and *GhFH30* expression is increased in response to high temperature and salt stress, and regulation of these genes would help to breed species that are more resistant to environmental stress [69]. Therefore, we screened four genes—*MtFH08*, *MtFH11*, *MtFH13*, and *MtFH14*—which were highly expressed under all three stress conditions, to be used as possible candidate genes. These candidate genes can be modified using gene editing tools in subsequent experiments to improve plant resistance to natural adversity.

## 5. Conclusions

In this study, we identified 18 *MtFH* genes and analyzed their chromosome distribution. The presence of segmental duplication and Ka/Ks in the species revealed the conservation of *MtFH* genes during evolution. The cis-acting elements of *MtFH* indicate that they are involved in the response to light and various hormones. Expression profiling of *MtFH* genes showed that *FH* genes contribute to *M. truncatula* growth and development, especially *MtFH17*, which was highly expressed in all six tissues. *MtFH08*, *MtFH13*, *MtFH11*, and *MtFH14* were highly expressed under the abiotic stresses. In addition, 22 miRNAs were predicted to aid in the regulation of *MtFH* genes at the transcriptional level. In summary, this study provides potential genes for breeding resistant crops. At present, the conclusions of this paper are mainly based on prediction, and future research needs to verify the predicted gene functions through specific experiments.

## Figures and Tables

**Table 1:** Basic information about the *Medicago truncatula FH* gene family.

Symbol	Gene ID	Chr	Start	End	aa	MW	pI	Instability Index	Aliphatic Index	GRAVY	Subcellular Localization
*MtFH01*	Medtr1g013800.1	Chr1	3233402	3244659	1778	194,744.01	5.84	60.86	79.26	−0.447	Nucleus
*MtFH02*	Medtr1g083260.1	Chr1	37051517	37055541	909	101,594.46	7.39	60.08	77.48	−0.547	Nucleus
*MtFH03*	Medtr2g082190.1	Chr2	34624278	34629796	860	95,004.64	8.58	45.73	77.21	−0.555	Chloroplast
*MtFH04*	Medtr2g089040.1	Chr2	37560923	37565770	847	95,007.30	8.34	47.33	79.22	−0.571	Chloroplast
*MtFH05*	Medtr3g037080.1	Chr3	13564187	13578034	1928	206,923.34	6.18	80.43	62.04	−0.618	Nucleus
*MtFH06*	Medtr3g078623.1	Chr3	35438534	35441319	689	77,181.65	8.42	48.24	79.83	−0.431	Nucleus
*MtFH07*	Medtr4g045670.1	Chr4	15509608	15513682	860	94,395.75	6.15	55.77	76.64	−0.529	Nucleus
*MtFH08*	Medtr4g081410.1	Chr4	31560742	31567076	984	106,691.00	8.52	55.67	77.73	−0.429	Nucleus
*MtFH09*	Medtr4g087890.1	Chr4	34465056	34468637	889	98,723.37	6.07	62.15	73.69	−0.523	Nucleus
*MtFH10*	Medtr4g095780.1	Chr4	39940525	39944800	857	94,815.78	8.88	48.34	77.58	−0.494	Nucleus
*MtFH11*	Medtr4g109040.1	Chr4	45203395	45215879	1576	171,050.56	6.40	69.23	65.86	−0.578	Nucleus
*MtFH12*	Medtr4g131020.1	Chr4	54634518	54646617	1198	133,396.00	8.38	58.39	77.82	−0.496	Nucleus
*MtFH13*	Medtr5g015690.1	Chr5	5489429	5494498	908	99,681.28	9.14	52.06	73.25	−0.599	Nucleus
*MtFH14*	Medtr5g026645.1	Chr5	10985497	10993656	1211	131,552.96	7.54	55.84	74.35	−0.422	Nucleus
*MtFH15*	Medtr5g036540.1	Chr5	15943741	15946226	797	87,539.55	9.21	57.17	80.36	−0.495	Nucleus
*MtFH16*	Medtr7g080920.1	Chr7	30828192	30832234	1012	112,425.62	6.89	66.84	72.23	−0.571	Nucleus
*MtFH17*	Medtr8g027995.1	Chr8	10323250	10328257	1071	117,088.69	8.62	69.42	73.44	−0.459	Nucleus
*MtFH18*	Medtr8g062830.1	Chr8	26282122	26285210	740	82,239.76	9.02	49.26	85.91	−0.492	Vacuole

aa: amino acid length; GRAVY: grand average of hydropathicity; pI: isoelectric point; MW: molecular weight; Chr: chromosome.

**Table 2:** Ratio of non-synonymous substitution (Ka) to synonymous substitution (Ks).

Gene 1	Gene 2	Ka	Ks	Ka/Ks	Purifying Selection	Duplicate Type
*MtFH04*	*MtFH07*	0.281891053	0.99328562	0.283796571	Yes	segmental
*MtFH10*	*MtFH13*	0.227763917	1.194490726	0.190678682	Yes	segmental

## Data Availability

The data are contained within the article.

## Supplementary Materials

The following supporting information can be downloaded at: https://www.mdpi.com/article/10.3390/genes16050555/s1, Table S1: Provide Synteny analysis of the MtFH and multispecies FH genes.
